# Epigenetic and mitochondrial regulation in kidney disease: emerging mechanisms and therapeutic opportunities

**DOI:** 10.3389/fimmu.2026.1910925

**Published:** 2026-10-12

**Authors:** Atul Rawat, Narayan Prasad, Chandan Komalkumar, Harshit Singh, Saurabh Chaturvedi, Sultan Ahmed, Brijendra Kumar, Ravi Shankar Kushwaha

**Affiliations:** 1 Department of Nephrology, Sanjay Gandhi Post Graduate Institute of Medical Sciences (SGPGIMS), Lucknow, India; 2 Department of Pharmacology, Faculty of Pharmacy, Sri Adichunchanagiri College of Pharmacy, Adichunchanagiri University, Mandya, India

**Keywords:** acute kidney injury, chronic kidney disease, diabetic kidney disease, epigenetics, immunometabolism, mitochondrial dysfunction, mitophagy, non-coding RNA

## Abstract

Renal fibrosis is an important pathogenic characteristic in the course of chronic kidney disease, driven by intricate interactions between mitochondrial failure and epigenetic control. The kidney is highly energy dependent and hence particularly vulnerable to mitochondrial disturbances such as decreased oxidative phosphorylation, mitochondrial reactive oxygen species (ROS) overproduction, reduced ATP production and mitochondrial DNA damage. Such changes induce oxidative stress and inflammatory reactions leading to tubular damage and fibrotic remodeling. Epigenetic processes such as DNA methylation, histone modifications and non-coding RNAs influence gene-expression programmes linked to inflammation, death, epithelial plasticity and extracellular-matrix deposition. Indeed, recent research indicates that there is reciprocal crosstalk between mitochondrial metabolism and epigenetic regulation, with metabolites such as NAD+, acetyl-CoA, α-ketoglutarate, succinate, fumarate and lactate, as well as ROS signaling, as mediators of this crosstalk. This interaction may also enhance inflammatory and fibrotic systems that drive chronic kidney disease progression. This mito-epigenetic axis has emerged as a therapeutic target. Potential strategies to mitigate renal fibrosis include SIRT1/PGC-1α signaling, AMPK activation, NRF2 antioxidant responses, epigenetic regulators (e.g., HDACs and DNMTs), and mitochondria-targeted antioxidants. Hence, this review analyses evidence according to illness, renal cell type, immunological state and translational maturity rather than considering mitochondrial and epigenetic alterations as uniform whole-kidney processes.

## Introduction

The prevalent incidence of renal disorders presents a persistent challenge in global healthcare, contributing significantly to morbidity, death, and treatment expenses worldwide. Kidney disease affects approximately ~850 million individuals globally. Recent global burden of Disease analyses further illustrates the mortality burden, estimating approximately 1.53 million deaths and 44.45 million disability-adjusted life years attributable to CKD in 2021 (1, 2). The economic consequences are similarly substantial: an international microsimulation across 31 countries and regions estimated annual direct healthcare costs for diagnosed CKD and kidney replacement therapy at approximately US$372.0 billion in 2022, projected to increase to US$406.7 billion by 2027. CKD is strongly associated with cardiovascular events, metabolic conditions, and premature mortality (3, 4). Furthermore, acute kidney injury (AKI) remains a critical clinical concern due to its elevated rate of in-hospital fatalities and its established involvement in ongoing renal impairment and the advancement of chronic kidney disease (5). Furthermore, diabetic kidney disease (DKD), the predominant etiology of end-stage renal disease (ESRD), has become more widespread owing to the rising population of individuals with diabetes and obesity (6). Notwithstanding advancements in therapeutic strategies such as renin-angiotensin system inhibition, SGLT2 inhibitors, and non-steroidal mineralocorticoid receptor antagonists (MRAs; e.g., finerenone), certain patients continue to have progressive nephron deterioration and renal fibrosis (7, 8).

Mitochondrial impairment has been identified as a significant contributor to renal injury owing to the organ’s elevated metabolic demands. The proximal tubule cells are rich in mitochondria and utilize oxidative phosphorylation for the transport of ions, absorption of solutes, and maintenance of tubular balance (9). Consequently, any breach in mitochondrial integrity swiftly results in malfunction among the renal cells. Current evidence strongly suggests that mitochondrial dysfunction is not merely a consequence of renal impairment but also plays a role in tissue remodeling and the deterioration of kidney function (10). Mitochondrial irregularities observed in renal disease encompass dysfunction of the electron transport chain (ETC), heightened production of mitochondrial reactive oxygen species (mtROS), mitochondrial depolarization, changes in mitochondrial dynamics, impaired fatty acid metabolism, and mtDNA damage (11).

Oxidative stress serves as a crucial molecular link between mitochondrial impairment and renal illness. Under typical conditions, reactive oxygen species produced by mitochondria contribute to redox signaling and cellular adaptability. In the case of constant metabolic stress, hyperglycemia, ischemia, hypoxia, and inflammatory stimuli lead to the excessive release of electrons from the respiratory chain, which causes an imbalance in mitochondrial ROS generation and subsequent macromolecular damage caused by oxidants (12). The continuous production of mtROS results in the oxidation of proteins, lipids, and nucleic acid, leading to the injury of podocytes, apoptosis of tubular cells, and dysfunction of the endothelium and glomerulosclerosis (13). mtDNA fragments, when released into the cytosol due to mitochondrial dysfunction, act as DAMPs, stimulating innate immune responses like cGAS-STING signaling and NLRP3 inflammasome activation (14). Additionally, inflammation stimulates pro-fibrotic signaling pathways such as NF-κB and TGF-β/Smad signaling pathways, contributing to renal fibrosis and CKD progression (15).

Equally important pathways regulating mitochondrial quality control play a critical role in modulating renal cell fates under conditions of stress and injury. Mitochondria are constantly exposed to fusion-fission events which are essential in maintaining mitochondrial structural and functional integrity, metabolic versatility, and stress resistance. Proteins involved in mitochondrial fusion such as MFN1, MFN2, and optic atrophy protein 1 (OPA1) protect mitochondrial structure and respiratory performance, while excessive mitochondrial fission by dynamin-related protein 1 (DRP1) leads to fragmentation, impairment of energy production capacity, and apoptosis (16). The process of targeted elimination of mitochondria via PINK1/Parkin-dependent autophagy is crucial in maintaining mitochondrial homeostasis. Defective mitophagic activity causes accumulation of defective mitochondria, reactive oxygen species, ATP depletion, and inflammation in patients with either AKI or CKD (17). Importantly, recent evidence suggests that AMPK, SIRT1/PGC-1α, and NRF2 signaling pathways are fundamental components responsible for mitochondrial biogenesis, redox modulation, and metabolic reprogramming upon renal injury (18).

Parallel progress in mitochondrial biology has also drawn attention to epigenetic regulation, yet another critical factor contributing to renal inflammation, fibrosis, and metabolic reprogramming. Epigenetic regulation refers to changes in gene function without any modification of the genomic structure and involves DNA methylation, histone modifications, chromatin remodeling, and non-coding RNA-mediated control (19). Metabolic and environmental factors like high glucose levels, oxidative stress, hypoxia, and uremic toxins lead to pronounced epigenetic remodeling, which contributes to adverse transcriptional programming observed in kidney diseases (20). DNMTs play an important role in suppressing gene expression by methylating CpG sites, while HATs, HDACs, and histone methyltransferases modulate chromatin access and promote inflammation through transcriptional activation of inflammatory genes (21). Overactivity of HDACs and modifications in histone acetylation patterns have been linked to TGF-β-mediated fibrosis, EMT, and persistent inflammation in CKD (22).

Recent observations have shown that the relationship between mitochondrial dysfunction and epigenetic remodeling has a strong connection rather than being mechanistically independent phenomena. Mitochondrial metabolites like acetyl-CoA, NAD+, α-ketoglutarate, succinate, and fumarate work as metabolic regulators of epigenetics and chromatin structure (23). Histone acetylation uses Acetyl CoA as a substrate, whereas the cofactor needed for SIRT1-dependent deacetylation is NAD +. Likewise, α-Ketoglutarate acts as a co-substrate for the TET family of DNA demethylation enzymes and Jumonji histone demethylation enzymes; on the other hand, succinate accumulation interferes with the function of these enzymes (24).

Together, all of these results demonstrate the existence of an interactive network between mitochondria and epigenetics that bridges metabolic stress to prolonged inflammation and fibrosis associated with renal disease. Elucidating the manner in which mitochondrial damage impacts epigenetic reprogramming, as well as epigenetic reprogramming that impacts mitochondria, may be key to unraveling progressive renal pathology and formulating better therapies. The integrated mito-epigenetic crosstalk underlying kidney disease is illustrated in **Figure 1**.

**Figure 1:**
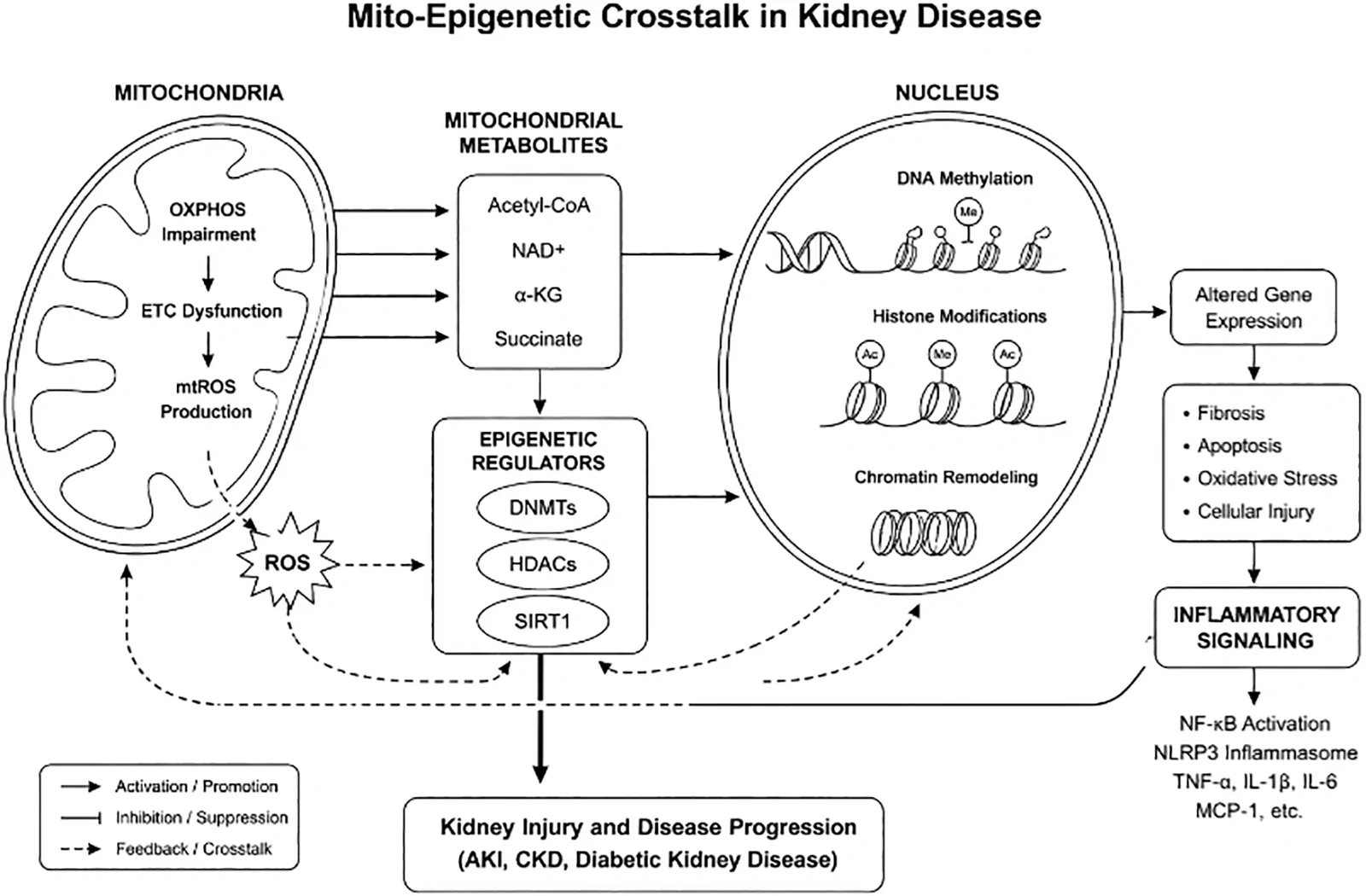
Integrated mito-epigenetic crosstalk in kidney disease.

The reciprocal interaction between mitochondria and epigenetics has been demonstrated to play an important role in AKI, CKD, DKD, and progressive renal fibrosis. Metabolic memory and mitochondrial ROS production due to chronic hyperglycemia contribute to persistent inflammation and profibrogenic signaling events, despite the normalization of blood glucose levels in DKD (25). The abrupt depolarization of mitochondria with rapid epigenetic reprogramming, associated with AKI caused by ischemia-reperfusion injury, determines the subsequent recovery, resolution of inflammation, and the maladaptive response after injury (26). Although considerable progress has been achieved, some critical issues are still not fully elucidated, such as which factor, mitochondria or epigenetics, initiates the cascade, the differences in mitochondrial-epigenetic signaling in different renal cells, and the reversibility of pathological epigenetic modifications in late kidney damage.

Considering the growing knowledge about mitochondria-related metabolism, oxidative stress, and epigenetics in nephrology, systematic assessment of the relationship between mitochondria and epigenetics has gained increasing relevance in terms of both its underlying mechanisms and translational aspects. While mitochondrial dysfunction and epigenetics have been traditionally studied separately, integrative studies of the interaction between these processes and potential treatment strategies remain relatively scarce. Therefore, this review explores the relationship between mitochondrial dysfunction and epigenetics in renal disease pathogenesis, focusing on oxidative stress, mitochondrial dynamics, mitophagy, inflammation, and metabolites-induced changes in chromatin structure. Potential treatment strategies involving the modulation of AMPK, SIRT1/PGC-1α, NRF2, HDAC, DNMT, and mitochondrial quality control systems are also considered (27, 28).

## Conceptual framework: mitochondrial metabolites as epigenetic couplers

Mitochondrial metabolism influences the epigenome because several chromatin-modifying enzymes require metabolites as substrates or cofactors. Acetyl-CoA supplies acetyl groups for histone acetyltransferases; NAD+ supports sirtuin deacetylases; α-ketoglutarate supports TET DNA demethylases and Jumonji histone demethylases; and succinate and fumarate can inhibit α-ketoglutarate-dependent demethylation (23, 24, 29, 30). Lactate can also participate in lysine lactylation, linking glycolytic reprogramming to chromatin regulation in inflammatory and fibrotic settings. The principal metabolite centered interfaces linking mitochondrial dysfunction to epigenetic regulation are summarized in **Table 1** and overall metabolite-centered mito-epigenetic framework is illustrated in **Figure 2**.

**Table 1:** Metabolite-centered interfaces linking mitochondrial dysfunction to epigenetic regulation.

Metabolic signal	Epigenetic interface	Renal disease context	Likely phenotype	Evidence interpretation
NAD+ depletion	Reduced sirtuin deacetylation	AKI, aging kidney, CKD	PGC-1α suppression, impaired biogenesis, inflammation	Intervention-supported in experimental AKI; clinical translation emerging (31, 32)
Acetyl-CoA availability	Histone acetylation substrate	DKD, fibrosis, metabolic stress	Inflammatory/fibrotic enhancer activation	Mechanistic metabolism-epigenetic link; kidney-specific causality incomplete (33)
α-Ketoglutarate	TET/Jumonji cofactor	Hypoxia, fibrosis, metabolic reprogramming	DNA/histone demethylation capacity	Strong biochemical basis; disease-specific renal validation evolving (24)
Succinate/fumarate	Demethylase inhibition	Fibrosis, hypoxia-like signaling	Profibrotic chromatin and EMT-like programs	Intervention-supported in non-renal models; renal extrapolation requires caution (33, 34)
mtROS/mtDNA	NF-κB and innate immune chromatin activation	AKI, CKD, DKD	Inflammasome activation, cytokines, fibrosis	Experimental support for cGAS-STING/NLRP3 links (14, 35, 36)

**Figure 2:**
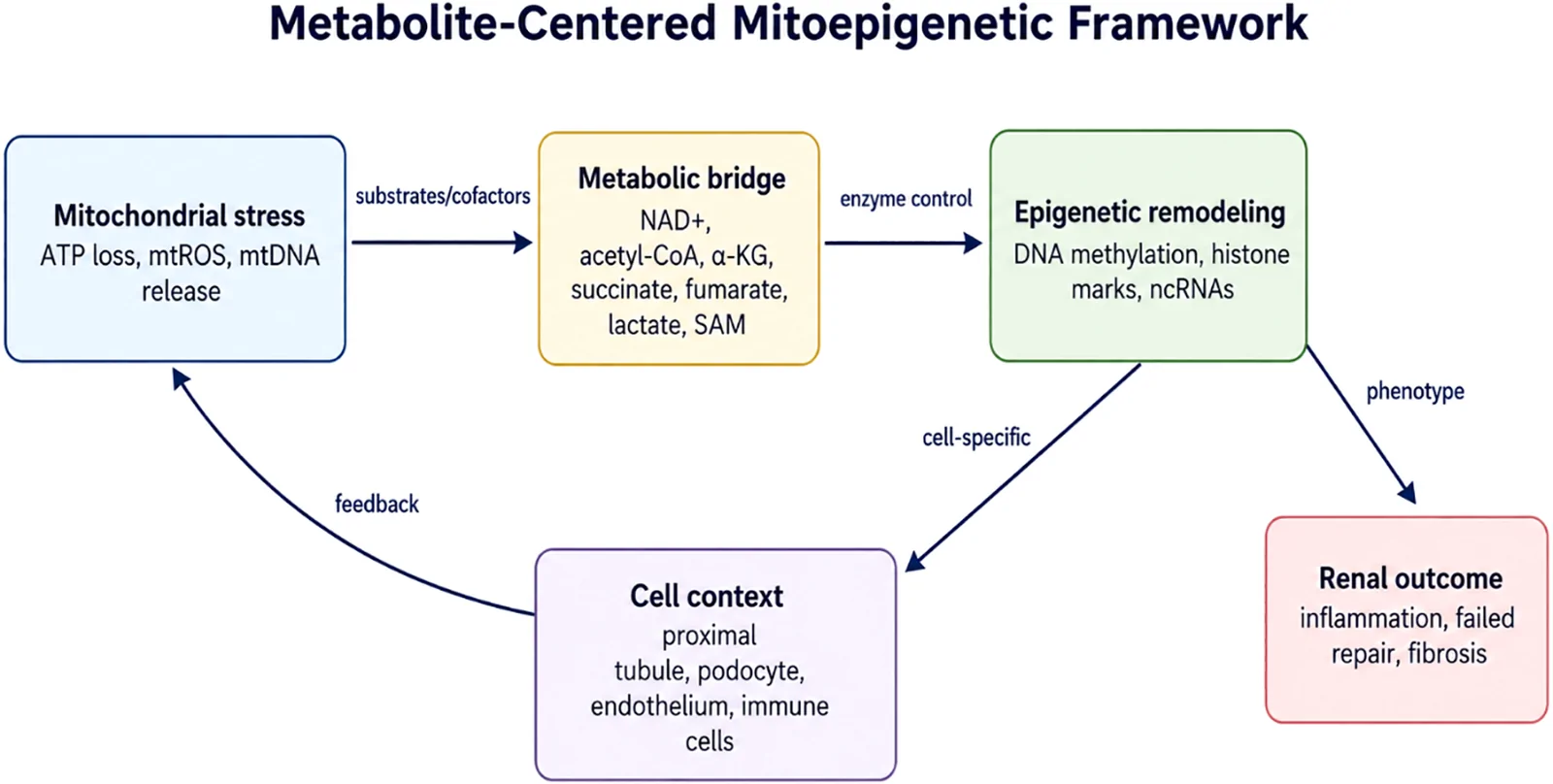
Metabolite-centered framework linking mitochondrial dysfunction, epigenetic remodeling, renal cell context, and progressive inflammation/fibrosis.

The direction of causality differs among models. Intervention-based studies support causal roles for selected mitochondrial-quality-control, SIRT1/PGC-1α, DNMT, HDAC, inflammasome, and DAMP-sensing pathways, whereas many human tissue and omics studies remain associative (31, 37–40). Accordingly, causal language is reserved for intervention-supported findings, while human correlations are described as associations or candidate mechanisms.

## Oxidative stress and reactive oxygen species production

Oxidative stress is regarded as one of the earliest and most enduring characteristics of renal pathologies. The renal tubular epithelial cells have a high-energy demand and rely heavily on mitochondrial oxidative phosphorylation as a source of energy. This implies that any disruption in the respiratory function of mitochondria will cause serious damage to renal cellular homeostasis. In scenarios of pathophysiological states such as hyperglycemia, ischemic-reperfusion injury, hypoxia, and nephrotoxic insults, increased electron leakage from ETC complexes I and III results in an overproduction of mtROS (including superoxide anions and hydrogen peroxide) (41).

Continuous production of mtROS causes oxidative damage to lipids, proteins, and nucleic acids and consequently leads to a decrease in the stability of cell membranes, enzyme function, and the genomic stability. mtDNA is especially vulnerable due to the proximity of the organelle to the electron transport chain and lack of protection by histones. Oxidative stress in mtDNA also results in increased inflammatory responses via DNA-dependent pathways and inflammasome signaling (42). DKD results from high glucose levels that stimulate production of mtROS, resulting in podocytopathy, renal tubular cell damage, and glomerulosclerosis (43).

On the other hand, antioxidant defense mechanisms become increasingly compromised in the course of progression of renal diseases. The nuclear factor erythroid 2-related factor 2 (NRF2), an essential transcription factor involved in cellular antioxidant responses, stimulates the expression of protective genes like HO-1, SOD, glutathione peroxidase, and NAD(P)H quinone oxidoreductase 1 (NQO1). In case of sustained oxidative and inflammatory stress, NRF2 signaling may be repressed, further increasing the redox imbalance and mitochondrial damage (44). Research on CKD patients and experimental models has revealed that reduced NRF2 function is associated with rapid fibrosis, endothelial dysfunction, and mitochondrial energy metabolism impairment (45). However, despite the protective role of NRF2 activation seen in animal experiments, its clinical application is complicated, as shown by cardiovascular toxicities reported in bardoxolone methyl trials (46). The major mitochondrial dysfunction and oxidative-stress pathways involved in kidney injury are summarized in **Figure 3**.

**Figure 3:**
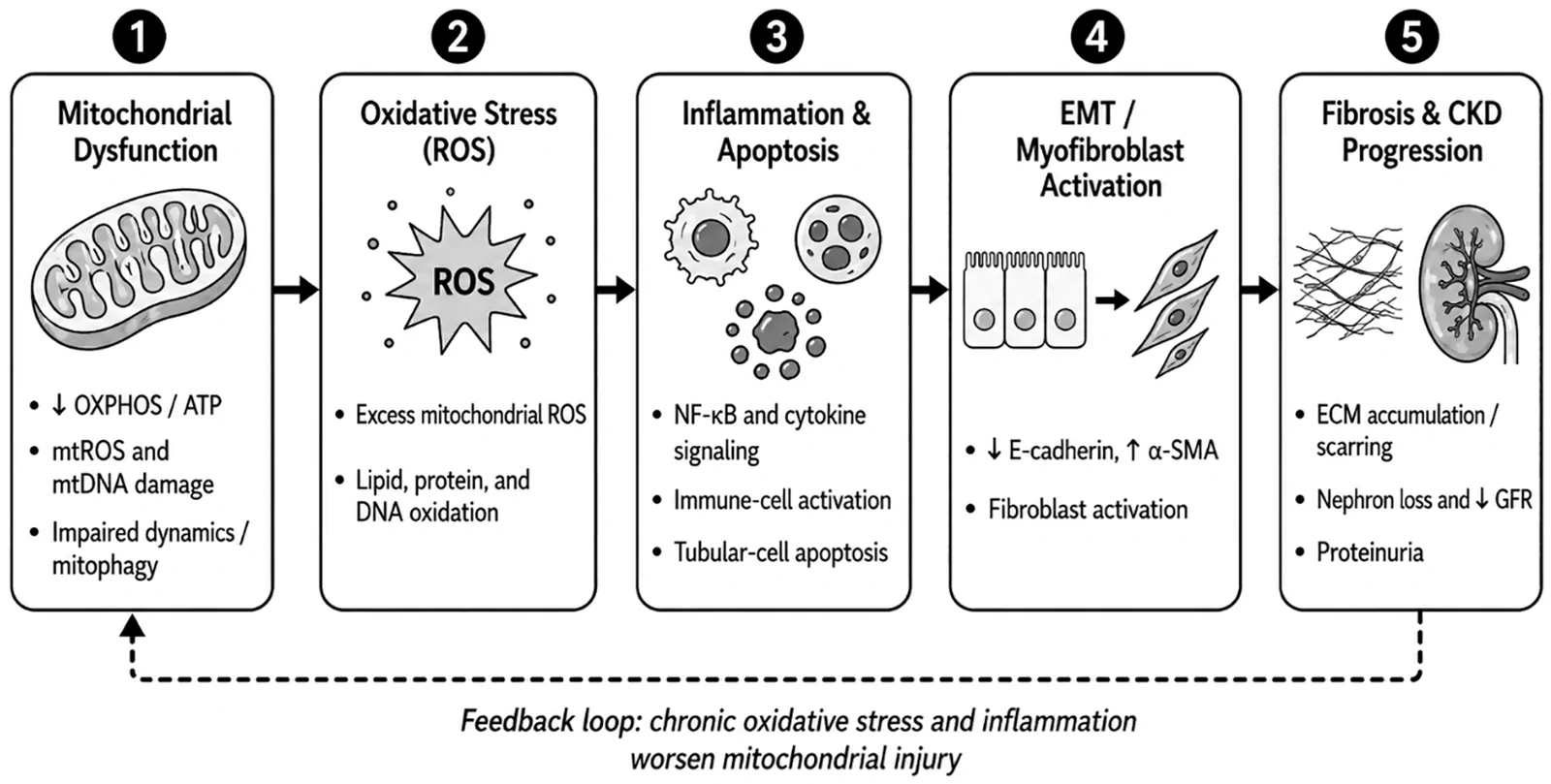
Mitochondrial dysfunction and oxidative stress pathways in kidney injury.

## Inflammation and inflammasome activation

The chronic nature of inflammation is one of the key hallmarks of progressive kidney disease. This chronic state of inflammation forms an important pathway that connects oxidative stress and tissue remodeling mechanisms. Damage from oxidative stress and mitochondria induce the production of pro-inflammatory pathways, especially the NF-κB pathway. This pathway plays an important role in the transcription process of cytokines, chemokines, and adhesion molecules for renal inflammation (47). The activation of the NF-κB pathway leads to an increased production of TNF-α, IL-1β, IL-6, and MCP-1 (48).

In addition to the role of NF-κB in signaling, mitochondrial dysfunction also participates in the mechanism of NLRP3 inflammasome activation. ROS from mitochondria, oxidation of mtDNA, lack of ATP, and potassium release together activate the assembly of NLRP3 inflammasome complexes, leading to caspase-1 activation and the processing of IL-1β and IL-18 (34). Inflammation caused by activation of the inflammasome leads to injury in tubular epithelial cells, endothelial cells, podocytes, and renal fibrosis. The effect of inflammasome activation can be blocked using either genetic or pharmacological interventions.

An inflammatory environment is established and enhanced in pathologic kidneys through epigenetic means as well. DNMTs and HDACs control the transcription of inflammatory genes through the regulation of chromatin access and cytokine expression (49). Enhanced HDAC signaling has been shown to sustain NF-kB activity and inflammation in cases of CKD and renal fibrosis (50). Non-coding RNAs, including the microRNAs, miR-21, miR-155, and miR-34a have been shown to affect inflammation and mitochondrial processes in renal pathologies (51). Additionally, noncoding RNAs like MALAT1 and NEAT1 have been seen to regulate inflammasomes and oxidative stress (52).

Inflammation is also associated with derangement of the microvasculature of the kidney and exacerbation of hypoxia within the tissues. Decreased availability of oxygen hinders mitochondrial metabolism, facilitates ROS generation, and activates the transcription factor HIF-1α, thereby promoting the metabolic shift to glycolysis and fibrogenesis (53).

## Renal immune microenvironment and mito-epigenetic reprogramming

The renal immune microenvironment includes tissue-resident leukocytes, recruited immune cells, and intrinsic renal cells that acquire immune-like functions during injury. Mitochondrial damage can release mtDNA, ATP, oxidized lipids, and other DAMPs that activate cGAS-STING, Toll-like receptors, and the NLRP3 inflammasome, linking renal metabolic stress to innate and adaptive immune activation (14, 19, 48).

Kidney-resident macrophages and recruited monocyte-derived macrophages have overlapping but non-identical functions. The conventional M1/M2 dichotomy is insufficient because single-cell studies identify inflammatory, interferon-responsive, lipid-associated, phagocytic, reparative, and matrix-remodeling states. SPP1-expressing macrophages are associated with fibrotic cell-cell communication in chronic kidney disease, whereas TREM2-associated states appear context-dependent: protective effects have been reported during AKI-to-CKD transition, but profibrotic signaling has also been demonstrated in obstructive nephropathy (54–58).

Adaptive immunity also influences injury and repair. CD4+ T-cell subsets can amplify or restrain renal inflammation; Th17-associated cytokines promote chemokine production and neutrophil recruitment, whereas Foxp3+ regulatory T cells can suppress innate inflammation and support recovery in ischemia-reperfusion models (59). CD8+ T-cell effects vary among transplantation, autoimmune nephritis, infection, sterile AKI, and metabolic CKD, so they should not be described as uniformly pathogenic.

Renal dendritic cells sense tissue-derived danger signals and connect innate sensing with antigen presentation. Albumin and other proteins encountered during injury can be internalized and proteasomally processed by renal dendritic cells, while maturation state and cytokine context determine whether antigen presentation promotes effector responses or tolerance (60). Direct kidney-specific evidence linking individual mitochondrial metabolites to stable dendritic-cell epigenetic states remains limited.

Neutrophils generate ROS, proteases, and neutrophil extracellular traps (NETs) during ischemic, septic, autoimmune, and crystal-associated injury. Excessive NET-associated DNA, histones, and granular proteins can amplify endothelial injury, coagulation, complement activation, and fibrosis, but their contribution differs among renal diseases (61, 62).

Lactate and protein lactylation provide an emerging metabolic-epigenetic bridge. Experimental CKD studies link macrophage H4K12 lactylation to phenotype remodeling and fibrosis, while sepsis-associated AKI studies connect macrophage HMGB1 lactylation with NET formation (61, 63). These findings support context-specific mechanisms but do not establish macrophage lactylation as a universal driver of inflammatory memory.

Together, mitochondrial DAMP sensing, macrophage-state transitions, dendritic-cell antigen presentation, T-cell balance, and NET-associated injury form a dynamic network rather than a linear cascade. The therapeutic implication is stage- and cell-state-specific modulation of immune metabolism and epigenetic persistence rather than broad, non-selective immunosuppression.

## Apoptosis and nephron loss

Impaired mitochondrial function is another key factor in inducing cell apoptosis associated with kidney disorders. Impaired mitochondrial membrane potential leads to an increase in mitochondrial permeability transition pore opening, release of cytochrome c in the cytosol, and activation of intrinsic apoptotic pathways (64). The regulation of these mechanisms is strictly controlled by the ratio of pro-apoptotic and anti-apoptotic factors, such as Bax and Bcl-2 proteins. An incresed Bax/Bcl-2 ratio in AKI and DKD promotes mitochondrial outer membrane permeabilization, which results in caspase-9 and caspase-3 activation (65).

Apoptosis of tubular epithelium plays an important role in the reduction of nephrons and impaired renal function. Hyperactive apoptosis affects the integrity of the epithelial lining, inhibits regeneration of the tubules, and increases the recruitment of inflammatory cells. Apoptosis of podocytes is even more harmful as podocytes have poor regenerative capabilities. The absence of podocytes damages the glomerular filtration barrier and promotes proteinuria and glomerulosclerosis (66).

Epigenetic regulation significantly influences apoptotic signaling in renal cells. Histone acetylation and DNA methylation affect the transcription of apoptosis-related molecules, while SIRT1 can exert anti-apoptotic effects through p53 deacetylation and redox regulation. Reduced NAD+ availability after kidney injury can impair SIRT1 activity and increase susceptibility to mitochondrial dysfunction and apoptosis (67). Kidney-specific experimental studies also implicate miR-34a in tubular and podocyte injury, although its effects vary with disease model and cellular context.

## Fibrosis and progressive renal remodeling

Renal fibrosis is a shared terminal pathologic event in virtually all progressive kidney disorders, which involve the excessive ECM deposition, activation of fibroblasts, and destruction of nephrons. Among fibrogenic pathways, the TGF-β/Smad pathway is the principal profibrotic pathway in progressive renal disease (68–70). Activation of the TGF-β signaling pathway drives EMT, myofibroblast differentiation, collagen deposition, and altered regulation of MMPs, which eventually leads to fibrotic scar formation and progression of chronic kidney disease. While the initial activation of the repair mechanism might be beneficial, continuous TGF-β stimulation leads to permanent fibrosis.

Impaired mitochondrial function is a key mediator of fibrosis via reduced ATP synthesis, enhanced oxidative stress, and dysfunctional fatty acid metabolism. Metabolic failure in tubular epithelial cells leads to the development of profibrotic features including enhanced release of cytokines, chemokines, and extracellular matrix proteins (71). The impairment in fatty acid metabolism, especially in proximal tubular epithelial cells, is increasingly recognized as a major cause of tubular dedifferentiation and fibrogenesis in CKD (35). In parallel, defective mitophagy and chronic mitochondrial fragmentation contribute to ROS production and inflammation, ultimately facilitating fibrotic signaling (72). Moreover, experimental data support the notion that chronic mitochondrial dysfunction mediates sustained activation of fibroblasts and deposition of extracellular matrix proteins via metabolic and redox-regulated gene expression (73).

Epigenetic remodeling is also crucial for the maintenance of transcriptional programs responsible for fibrosis in progressive renal damage. Condensation of chromatin through HDAC-mediated histone deacetylation, abnormal histone methylation, and pathologic DNA hypermethylation result in continuous activation of genes responsive to TGF-β as well as fibrotic pathways (74). Histone methyltransferase EZH2, an oncogene involved in fibroblast activation, fibrogenesis, and kidney scarring, achieves its effect by trimethylation of histone H3 lysine 27 (H3K27me3) and repressing antifibrotic genes (75). In addition, miRNA and long noncoding RNA (lncRNA) regulators of transcription such as miR-21 and TUG1 affect the signaling, metabolic processes, and inflammation in CKD and DKD (76). Taken together, epigenetic remodeling plays a functional role beyond its association with fibrosis and stabilizes pathological transcriptional programs contributing to renal damage.

Mitochondrial metabolites are also involved in controlling the epigenetics of fibrosis directly. The buildup of succinate can lead to the inhibition of histone and DNA demethylases by interfering with their dependence on α-ketoglutarate, resulting in prolonged gene expression in fibrosis. On the other hand, availability of acetyl-CoA impacts histone acetylation related to fibrosis or inflammation.

Collectively, oxidative stress, inflammation, apoptosis, and fibrosis form an integrated pathological network that contributes to progressive renal damage in AKI, CKD, DKD, and renal fibrosis. Mitochondrial dysfunction and disrupted epigenetic regulation can reinforce one another, sustaining metabolic failure, inflammation, and maladaptive tissue repair. Better definition of this mito-epigenetic network may reveal therapeutic opportunities that are specific to disease stage and cell type.

## Disease-specific mitoepigenetic patterns

Kidney diseases share oxidative stress, inflammation, regulated cell death, and fibrosis, but they differ in initiating insult, temporal window, dominant renal cell population, immune context, and therapeutic opportunity. Disease-stratified interpretation reduces overgeneralization and clarifies whether mitochondrial stress is an early driver, a marker of established damage, or part of maladaptive repair. Disease and cell type specific stratification of the mito-epigenetic axis, the major mitochondrial and epigenetic alterations across representative kidney diseases, and the corresponding disease-stratified mitochondrial, epigenetic, immune, and fibrotic mechanisms are summarized in **Figures 4** and **5** and **Table 2**.

**Figure 4:**
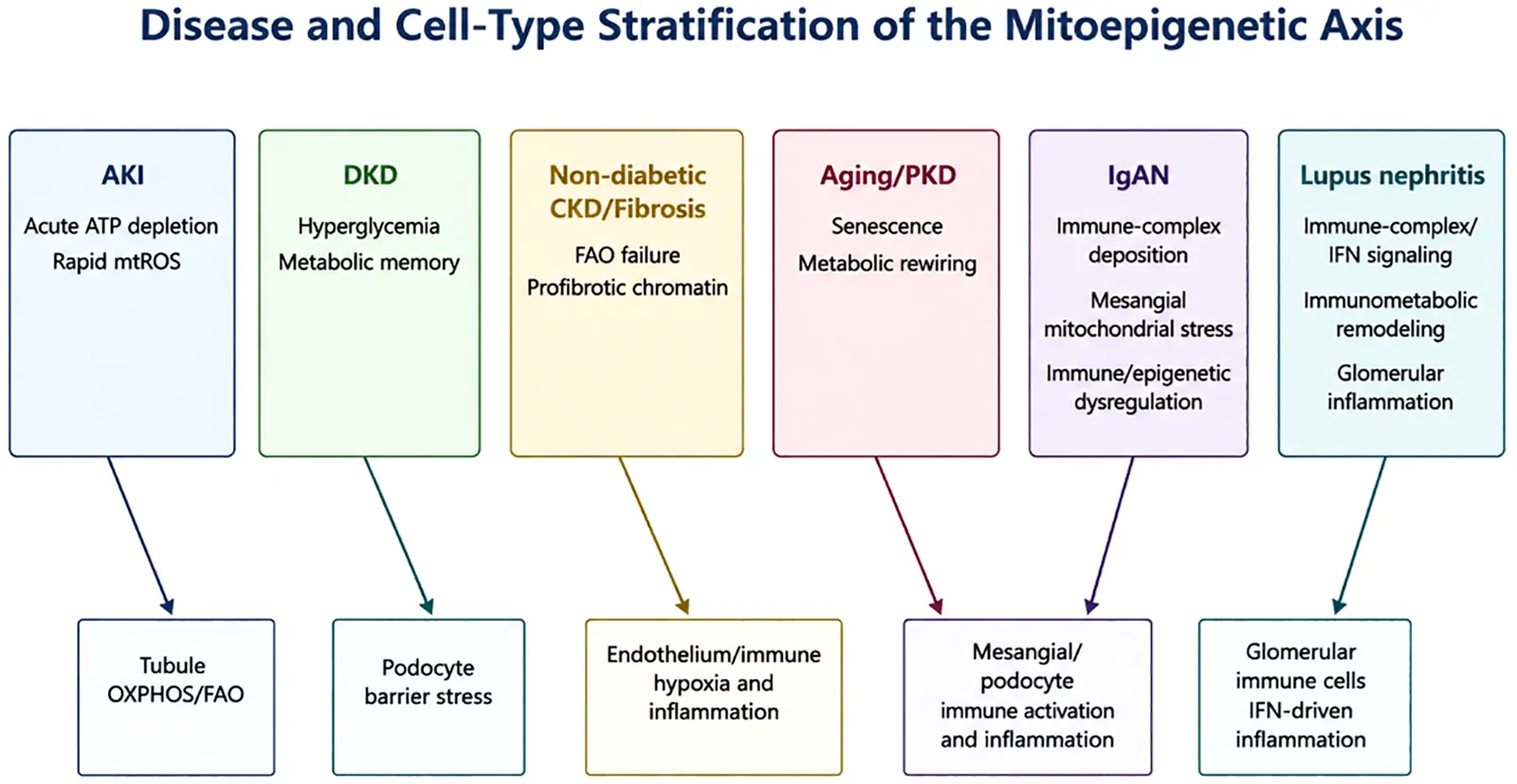
Disease and cell-type stratification showing why the Mito epigenetic axis should not be interpreted as a single whole-kidney mechanism.

**Figure 5:**
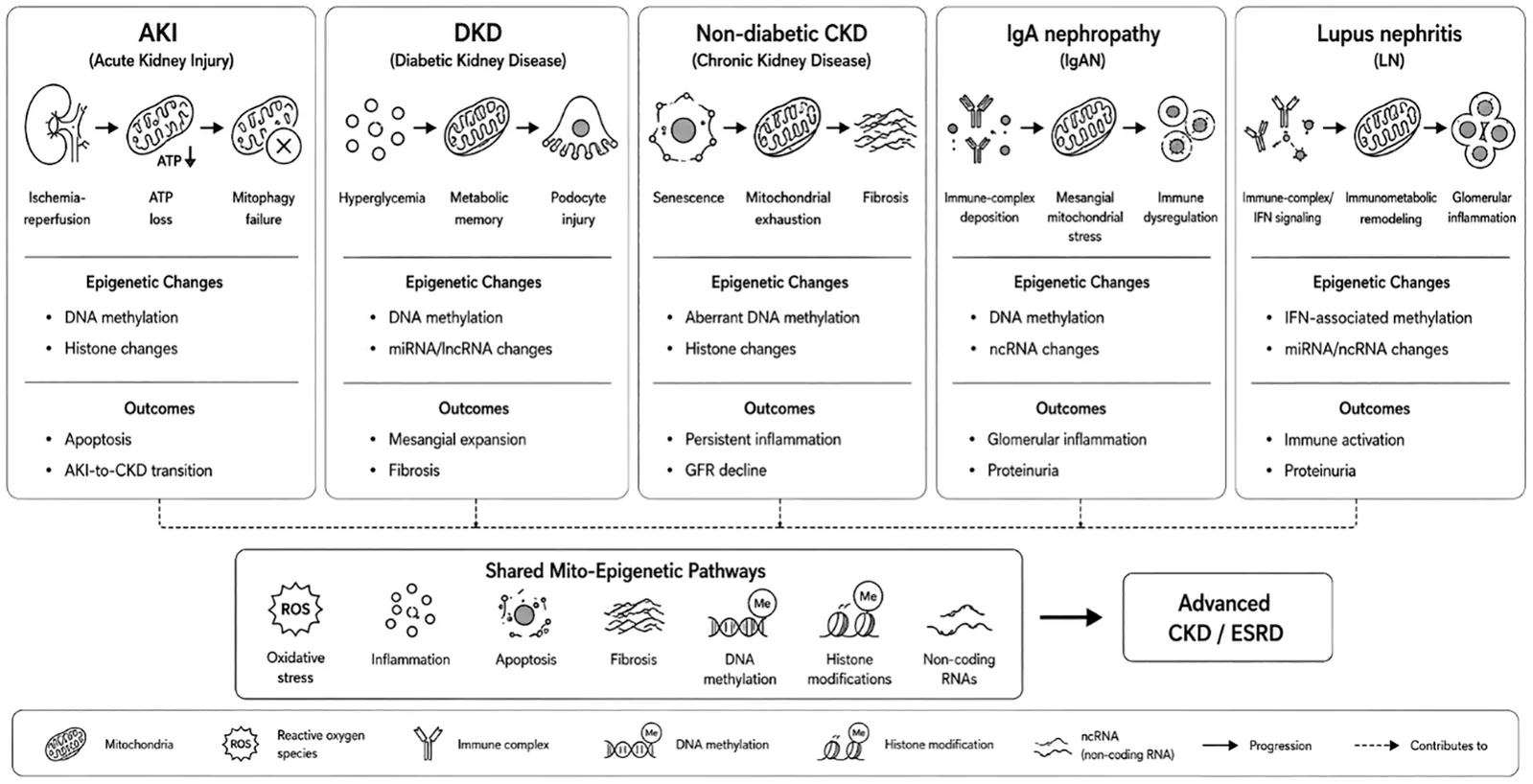
Major epigenetic mechanisms in kidney disease.

**Table 2:** Disease-stratified mito epigenetic mechanisms.

Disease context	Temporal window and dominant cell stress	Mitochondrial state	Epigenetic or RNA pattern	Immune/fibrotic phenotype	Therapeutic implication
Ischemia/reperfusion AKI	Minutes to days; proximal tubules and endothelium	ATP depletion, membrane depolarization, Drp1-linked fragmentation, mtDNA release	Rapid stress-responsive chromatin changes; altered DNA methylation during repair	DAMP sensing, NLRP3/cGAS-STING activation, failed repair	Early mitochondrial stabilization and controlled mitophagy may be more relevant than late antifibrotic therapy (14, 16, 26, 37)
Diabetic kidney disease	Months to years; podocytes, tubules, mesangial and endothelial cells	Hyperglycemia-driven mtROS, impaired FAO, metabolic inflexibility	Metabolic memory; miR-21/miR-192/miR-34a; histone acetylation changes	Low-grade inflammation, albuminuria, matrix deposition	Therapy should pair metabolic control with targets that interrupt persistent epigenetic memory (20, 21, 25)
Progressive CKD/fibrosis	Chronic maladaptive repair; tubules, fibroblasts, immune cells	FAO failure, defective mitophagy, persistent ROS	HDAC activation, EZH2-mediated repression, profibrotic methylation	Myofibroblast activation, macrophage persistence, ECM accumulation	Cell-selective epigenetic or metabolic delivery is critical because systemic HDAC/DNMT blockade is risky (68, 71, 74, 75)
Aging kidney	Long-term senescence; tubules, endothelium, immune cells	mtDNA mutations, reduced biogenesis, NAD+ decline	Klotho methylation, senescence-associated chromatin changes	Sterile inflammation, reduced repair reserve, fibrosis susceptibility	NAD+ restoration and senolytic/immunometabolic strategies need renal safety validation (77, 78)
Polycystic kidney disease	Developmental and chronic cyst expansion; epithelial metabolic rewiring	Altered oxidative metabolism and mTOR-linked metabolic reprogramming	miRNA dysregulation and chromatin changes affecting proliferation	Cyst growth, inflammation, interstitial fibrosis	Metabolic-epigenetic interpretation is plausible but less mature than AKI/DKD evidence (79, 80)
IgA nephropathy	Chronic/relapsing; mesangial cells, podocytes, B/T cells	Altered mesangial respiration, mitochondrial dynamics and mtROS; MFN1-linked dysfunction	CD4+/B-cell DNA methylation changes; miRNA dysregulation including miR-23b	Mesangial proliferation, immune-complex inflammation, proteinuria, glomerulosclerosis	Mesangial mitochondrial stabilization combined with disease-specific immune/epigenetic modulation; clinical validation required
Lupus nephritis	Relapsing immune-complex nephritis; glomerular cells plus infiltrating T/B cells and myeloid cells	Context-dependent T-cell metabolic/mitochondrial remodeling; altered NAD+ metabolism	IFN-associated DNA hypomethylation; renal miRNA dysregulation	Immune-complex inflammation, IFN signaling, glomerular/tubular injury and fibrosis	Cell-state-specific immunometabolic and epigenetic targeting rather than generalized mitochondrial stimulation

## Primary glomerular diseases: IgA nephropathy and lupus nephritis

IgA nephropathy (IgAN) provides a distinct immune-complex-mediated glomerular context for mito-epigenetic regulation. In contrast to the predominantly tubular metabolic failure emphasized in progressive tubulointerstitial CKD, mesangial cells and adaptive immune cells are major pathogenic compartments in IgAN. Experimental and patient-derived evidence indicates altered mitochondrial respiration and mitochondrial homeostasis during IgAN progression. Recent mechanistic work has linked MYC/HRD1-dependent degradation of MFN1 to mitochondrial dysfunction, whereas altered mitochondrial respiration in mesangial cells has been associated with mesangial proliferation and inflammatory signaling (81). Epigenetic dysregulation is also evident: genome-wide studies have identified disease-associated DNA methylation changes in CD4+ T cells and CD19+ B cells, including methylation networks related to immune signaling and tubulointerstitial fibrosis (82). In addition, loss of miR-23b-3p produces an IgAN-like phenotype with dysregulated mucosal IgA synthesis, supporting a functional contribution of non-coding RNA regulation rather than a purely associative relationship (83). These findings suggest that the mito-epigenetic landscape of IgAN is shaped by the interaction between mesangial mitochondrial stress, immune-cell epigenetic programming and IgA-mediated inflammation.

Lupus nephritis (LN) represents another distinct glomerular setting in which mitochondrial metabolism, epigenetic programming and immune-cell activation intersect. The direction of mitochondrial remodeling is highly cell- and disease-state-dependent. Kidney-infiltrating T cells in experimental lupus can acquire metabolically suppressed states characterized by impaired mitochondrial function, whereas other studies demonstrate that NAMPT-dependent NAD+ biosynthesis supports glycolysis, mitochondrial respiration and IFN-γ production in pathogenic CD4+ T cells (84). Epigenetic abnormalities further distinguish renal lupus from generic CKD. Patients with renal involvement exhibit disease-specific DNA methylation changes, including enhanced hypomethylation of interferon-regulated loci, while renal-biopsy profiling has identified altered microRNA networks, including marked upregulation of miR-422a in active LN (85). Thus, LN illustrates how immune-cell metabolic state, interferon-associated epigenetic remodeling and local renal-cell injury can converge within the mito-epigenetic axis, emphasizing that mitochondrial abnormalities should not be assumed to have the same direction or functional significance across renal diseases.

## Cell-type specificity

Whole-kidney analyses can conceal divergent mitochondrial and epigenetic programs. Proximal tubules depend strongly on fatty-acid oxidation and oxidative phosphorylation; podocytes have limited regenerative capacity; endothelial cells connect hypoxia to microvascular and immune responses; fibroblasts integrate metabolic stress into matrix synthesis; and immune cells can acquire persistent transcriptional states (9, 15, 66, 71, 86). Renal cell-type-specific mito-epigenetic vulnerabilities and potential interventions are summarized in **Table 3**.

**Table 3:** Renal cell-type-specific mitoepigenetic vulnerabilities.

Cell type	Mitochondrial vulnerability	Epigenetic/RNA signal	Pathological phenotype	Candidate intervention
Proximal tubular epithelial cell	High OXPHOS/FAO demand; ATP loss and defective mitophagy	SIRT1/PGC-1α suppression, Klotho methylation, miR-21	Failed repair, cytokine release, fibrosis	PGC-1α/FAO restoration; targeted mitochondrial antioxidants (9, 31, 71)
Podocyte	mtROS compromises cytoskeleton and filtration barrier	miR-34a/miR-21 and apoptosis-linked transcription	Proteinuria and glomerulosclerosis	ROS control and apoptosis modulation (41, 66)
Endothelial cell	Hypoxia-sensitive mitochondrial signaling	Inflammatory enhancer activation and nitric oxide pathway changes	Capillary rarefaction, leukocyte recruitment	Endothelial-protective metabolic and anti-inflammatory strategies (53, 87)
Fibroblast/myofibroblast	Metabolic reprogramming supports matrix production	EZH2, HDAC activity, profibrotic methylation	ECM accumulation and scarring	Fibroblast-selective epigenetic modulation (39, 68, 75)
Immune cells	Immunometabolic shift toward inflammatory glycolysis/ROS	NF-κB chromatin priming, ncRNA regulation	Persistent inflammation and fibrosis amplification	Macrophage/T-cell immunometabolic targeting (19, 59)
Mesangial cell	Hyperglycemia-associated mtROS generation, impaired mitochondrial respiration, and metabolic reprogramming toward a matrix-producing phenotype	TGF-β-responsive miR-192 signaling, with associated miRNA and chromatin changes that reinforce profibrotic gene expression	Mesangial proliferation, increased collagen and fibronectin synthesis, mesangial matrix expansion, and progressive glomerulosclerosis	Reduction of mitochondrial ROS, restoration of metabolic homeostasis, and selective modulation of the TGF-β/Smad–miR-192 axis (20, 25, 41, 88)

In diabetic kidney disease, mesangial cells develop hyperglycemia-associated mitochondrial oxidative stress and metabolic reprogramming. These changes interact with TGF-β-responsive miR-192 networks to promote extracellular-matrix synthesis, mesangial expansion, and glomerulosclerosis. The relative contributions of mitochondrial dysfunction and persistent epigenetic programming remain stage-dependent and incompletely resolved (20, 25, 41, 88, 89).

In recent years, chromatin remodeling driven by oxidative stress has come to be considered an important mediator between mitochondrial impairment and chronic renal damage. The role played by reactive oxygen species in inflammation and the consequent modification of histone access, transcription factor binding, and enhancer function allows for continuous expression of inflammation and fibrosis-related genes even after removal of the initial trigger (90). In DKD, “metabolic memory”, which allows for past episodes of hyperglycemia to trigger sustained transcriptional events following correction of blood glucose levels, provides a notable example (21).

## DNA methylation

DNA methylation is another extensively studied epigenetic mechanism associated with kidney diseases. DNMTs, including DNMT1, DNMT3A, and DNMT3B, facilitate methyl group transfer to cytosine residues on CpG islands, typically resulting in transcriptional inhibition (91). There is growing evidence that abnormal methylation patterns play a significant role in renal fibrosis, inflammation, tubular epithelial damage, and maladaptive repair processes.

Hypermethylation-induced gene silencing is particularly pertinent in CKD development. The promoter region hypermethylation of Klotho, an anti-aging and cytoprotective protein, has been linked to increased oxidative stress, mitochondrial dysregulation, tubular senescence, and TGF-β-induced fibrosis. Methylation-induced repression of antioxidant and mitochondrial regulators also causes adaptive stress response dysfunction in renal tubular cells. On the other hand, hypomethylation of inflammatory and fibrotic regions contributes to chronic NF-κB and TGF-β signaling, thereby increasing cytokine secretion and extracellular matrix deposition (92).

Additional research findings also indicate that mitochondrial impairment has a direct impact on DNA methylation via metabolic control of epigenetic factors. α-Ketoglutarate is a cofactor for TET-mediated DNA demethylation, while succinate accumulation blocks TET enzymes and results in pathologic hypermethylation states (24). Thus, impaired mitochondrial metabolism can potentially lead to major changes in transcriptional pathways controlling fibrosis, apoptosis, inflammation, and mitochondrial function itself. Yet, whether such methylation alterations can be entirely reversed in late-stage CKD is still unknown.

## Histone modifications

The role of histone modifications includes control over the accessibility of chromatin and transcription through reversible modification of the histone tail region. Histone acetylation leads to increased transcription, while histone deacetylation promotes transcriptional repression and chromatin condensation (93). Histone acetylase enzymes, histone deacetylase enzymes, and histone methyltransferase enzymes have been implicated in kidney pathology due to their maladaptive inflammatory and fibrotic signaling pathways.

Increased HDAC activity is one of the most potent factors in renal fibrosis and EMT. Increased chromatin condensation caused by HDAC increases the action of TGF-β/Smad signaling pathways leading to transcription of collagen, fibronectin, and α-SMA, which results in myofibroblast proliferation and extracellular matrix synthesis (50). Additionally, H3K9ac has been observed to play a role in the activation of inflammatory genes in diabetic nephropathy, while the repressive H3K27me3 plays a critical role in fibrotic gene silencing and chromatin stability (94).

Sirtuin signaling serves to link histone modifications to the mitochondria’s metabolic pathways. The NAD+-dependent histone deacetylase, SIRT1, exhibits anti-inflammatory and antioxidant actions due to its ability to deacetylate NF-κB, p53, and PGC-1α (31). Inadequate NAD+ levels in CKD compromise SIRT1 action, exacerbating oxidative stress, mitochondrial malfunction, and vulnerability to apoptosis. Acetyl-CoA is responsible for histone modification since it serves as the substrate in the histone acetylation reaction catalyzed by HATs. This suggests that mitochondrial metabolism and chromatin modification are intricately linked. All of these findings support the notion that histone modifications act not just as end products of renal damage but also as active participants in the development and progression of kidney diseases.

## Non-coding RNAs

Non-coding RNAs contribute to renal mito-epigenetic signaling by regulating oxidative stress, mitochondrial bioenergetics, apoptosis, inflammation, and fibrosis. Their interpretation depends on disease model, cell type, validated target, and renal functional endpoint rather than on expression change alone (51, 76, 88).

miR-21, miR-192, miR-34a, miR-155, TUG1, MALAT1, and NEAT1 illustrate different levels of evidence. miR-21 and TUG1 have comparatively stronger intervention-based or mechanistic support, whereas many circRNA findings remain hypothesis-generating until target validation and renal functional endpoints are available (51, 76, 95). The major non-coding RNAs associated with renal mito-epigenetic regulation are summarized in **Table 4**.

**Table 4:** Non-coding RNAs linked to renal mito epigenetic regulation.

RNA	Disease/model	Target cell/context	Mitochondria-associated action	Epigenetic/regulatory role	Downstream phenotype	Evidence type
miR-21	DKD, CKD, Alport/fibrosis models	Tubules, fibroblasts, glomerular compartments	Suppresses metabolic pathways; associated with mitochondrial dysfunction	Post-transcriptional control of profibrotic signaling	TGF-β amplification, fibrosis, ECM deposition	Intervention-supported in animal models (51, 76, 95)
miR-192	Diabetic nephropathy	Mesangial and tubular contexts	Indirect metabolic stress association	TGF-β-responsive miRNA network	Matrix expansion and fibrosis	Experimental and associative (25, 88)
miR-34a	DKD/renal apoptosis contexts	Tubular cells and podocyte stress	Apoptosis and mitochondrial injury association	p53-linked regulatory circuit	Cell death and impaired repair	Experimental; renal specificity and direction vary by model (96)
miR-155	Inflammatory kidney injury	Immune and renal inflammatory compartments	Immunometabolic activation	Inflammatory transcript regulation	Cytokine production and macrophage activation	Experimental/associative (51)
TUG1	Diabetic nephropathy	Podocyte/tubular metabolic stress	Regulates mitochondrial bioenergetics	lncRNA control of transcriptional/metabolic networks	Bioenergetic preservation or dysfunction depending on expression	Mechanistic animal/cell evidence (97)
MALAT1/NEAT1	Inflammation and fibrosis models	Tubules and immune-interacting compartments	Oxidative stress and inflammasome association	lncRNA scaffolding/regulatory effects	Inflammation, fibrosis, cellular stress	Experimental; disease specificity incomplete (52)
circRNAs	Emerging renal disease studies	Cell-type dependent	Potential miRNA sponge effects on mitochondrial and fibrotic targets	Post-transcriptional regulatory hubs	Fibrosis/inflammation hypotheses	Emerging; direct renal functional validation limited

## Mitochondrial control in kidney disorders

Considering the unique metabolic requirements of renal tissue, mitochondria play an essential role in sustaining nephron functions. The oxidative phosphorylation pathway contributes significantly to ATP generation in renal tubular epithelial cells, and any slight aberration in mitochondrial bioenergetics leads to severe disruptions in tubular transport, ion balance, and cell survival (9).

## Mitochondrial bioenergetics

The mitochondrial oxidative phosphorylation pathway serves as the primary source of energy for renal tubular epithelial cells. Impaired electron transport chain activity causes ATP depletion, mitochondrial membrane depolarization, and bioenergetic dysfunction during kidney damage. AKI and DKD are associated with ischemic and hyperglycemic conditions that affect mitochondrial respiration and induce metabolic switching to glycolysis. Podocytes, with limited glycogen storage capacity, seem highly vulnerable to mitochondrial malfunction and oxidative stress.

Inadequate ATP levels can cause disruption of tubular polarity, ion transport function, cytoskeleton dynamics, and barrier functions, thus triggering nephron impairment and apoptosis. On the other hand, inadequate fatty acid catabolism can lead to lipid storage and fibrosis in CKD (71). Bioenergetic stress may also disrupt mitochondrial signaling and increase maladaptive transcription events, which have been linked to CKD-associated inflammation and fibrosis.

## Oxidative stress

Although excessive mtROS promotes renal injury and inflammation, low-level ROS signaling also contributes to physiological stress sensing and mitochondrial quality control; therefore, complete suppression of mitochondrial ROS may not be therapeutically desirable.

## Mitochondrial dynamics

The dynamics of mitochondria are controlled by the processes of fusion and fission, which help preserve mitochondrial integrity and plasticity in metabolism. The proteins involved in the fusion process include MFN1, MFN2, and OPA1, while the fission process is mediated by Drp1 (16).

Activation of pathological Drp1 triggers mitochondrial fragmentation, production of reactive oxygen species, cytochrome c leakage, and apoptotic signaling. A decrease in MFN2 level also disrupts mitochondrial-ER crosstalk and calcium regulation in kidney cells (41). Disruption of mitochondrial dynamics has been repeatedly linked with AKI, DKD, and renal fibrosis. However, it is unclear whether mitochondrial fission is always harmful since it could be part of an initial reaction aimed at eliminating damaged mitochondrial components prior to causing irreversible damage.

## Mitophagy

Mitophagy represents a pivotal quality control process of mitochondria that selectively eliminates dysfunctional mitochondria via autophagy. This process is regulated by the PINK1/Parkin pathway. Upon mitochondrial depolarization, PINK1 gets recruited to the mitochondrial outer membrane, where it recruits Parkin to induce ubiquitination and degradation of faulty mitochondria (14).

Mitophagy defects lead to the accumulation of dysfunctional mitochondria, generation of ROS, reduction in ATP levels, and activation of inflammasomes. Evidence from various experiments shows that impairment in PINK1/Parkin pathway enhances tubular cell damage and fibrosis, while mitophagy improvement alleviates kidney damage (37). In addition, chronic inflammation and epigenetic alterations may worsen the status of mitophagy, thus creating a feed-forward loop for mitochondrial damage and adaptive organ remodeling.

## Mitochondria and epigenetics

A growing body of literature suggests that there is a close interplay between mitochondria and epigenetic modifications to regulate the fate of cells in disease and repair processes. Instead of being two separate events, mitochondria and epigenetics interact with each other through metabolism and stress signaling pathways (23).

Mitochondrial biogenesis and oxidative metabolism are regulated in part through the AMPK-SIRT1-PGC-1α network. AMPK supports adaptation to energy stress, while SIRT1-dependent deacetylation of PGC-1α can promote mitochondrial biogenesis, antioxidant capacity, and fatty-acid metabolism (31, 98, 99). Disruption of this network is associated with ATP depletion, mitochondrial fragmentation, inflammation, and fibrotic remodeling in experimental kidney disease, but the relative contribution of each component varies by disease stage and cell type.

Epigenetic mechanisms can also influence mitochondrial biogenesis and metabolism by regulating genes involved in oxidative metabolism, mitochondrial dynamics, antioxidant defense, and mitophagy. HDAC-dependent repression of mitochondrial regulators and non-coding-RNA-mediated control of apoptosis or ROS production can further impair mitochondrial homeostasis (30). This feedback establishes an amplification loop in which mitochondrial dysfunction induces epigenetic changes that, in turn, worsen metabolic and organelle injury.

## Therapeutic opportunities

Therapeutic strategies targeting the mito-epigenetic axis include epigenetic modulators, mitochondrial protectors, metabolic regulators, and approaches directed at mitochondrial quality control; however, most remain supported predominantly by preclinical evidence (34). The therapeutic classes, their corresponding evidence gradient, and evidence-graded therapeutic strategies targeting the mito-epigenetic axis are summarized in **Figure 6** and **Table 5**.

**Figure 6:**
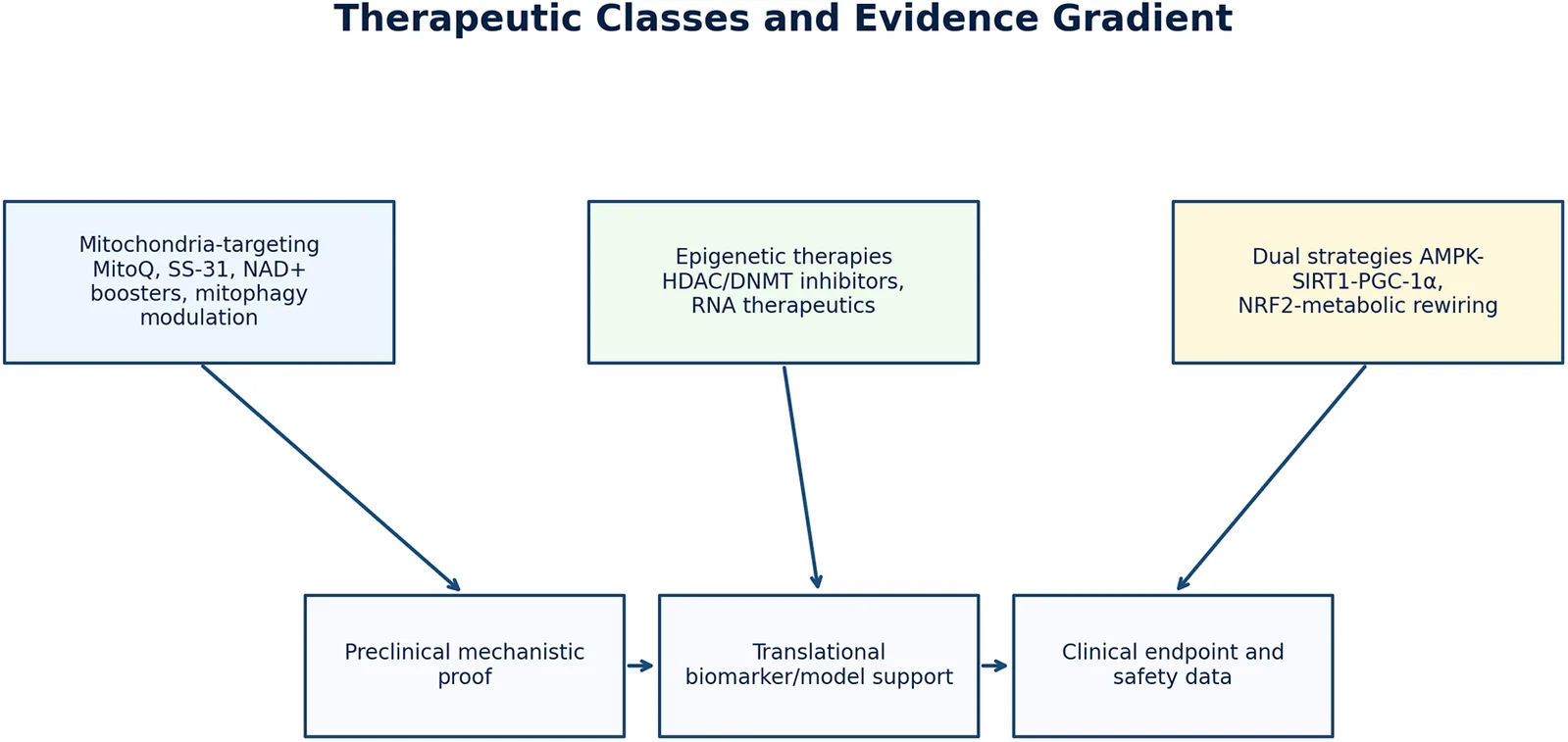
Therapeutic classes organized by primary target and evidence gradient.

**Table 5:** Evidence-graded therapeutic strategies targeting the mitoepigenetic axis.

Strategy	Class	Study type/model	Primary renal or mechanistic endpoint	Disease relevance	Key limitation/safety issue	Evidence interpretation
Metformin	Mitochondrial/metabolic	Clinical use in diabetes; renal mitoepigenetic benefits remain indirect/contextual	AMPK activation, metabolic adaptation, inflammatory suppression	DKD/CKD with diabetes	Dose adjustment, temporary interruption during acute illness, and avoidance in severe kidney dysfunction because of lactic-acidosis risk	Clinically used drug; kidney-specific mechanism remains mainly translational (100)
Resveratrol/SIRT1 activation	Dual mitoepigenetic	Cell and animal studies; limited clinical consistency	SIRT1-PGC-1α activation, NF-κB/p53 deacetylation	AKI, DKD, aging kidney	Bioavailability and inconsistent human efficacy	Promising preclinical mechanism; human renal efficacy is inconsistent (31, 90)
MitoQ	Mitochondria-targeting	Kidney-specific cell and animal studies	Reduced mtROS/lipid peroxidation	AKI, DKD, CKD	Long-term renal endpoint data limited	Preclinical renal evidence; durable clinical renal endpoints unavailable (101)
SS-31/elamipretide	Mitochondria-targeting	Preclinical ischemic kidney injury and mitochondrial models	Cardiolipin stabilization, cristae protection, respiration	AKI/CKD contexts	Disease-specific clinical renal benefit not established	Mechanistically strong preclinical evidence; renal clinical benefit not established (102)
HDAC inhibitors	Epigenetic	Cell/animal AKI, DKD, fibrosis models	Chromatin accessibility, TGF-β/Smad and inflammatory suppression	CKD/fibrosis/DKD	Isoform nonselectivity, systemic toxicity, off-target chromatin remodeling	Preclinical; clinical renal translation requires caution (22, 87, 103, 104)
DNMT inhibitors	Epigenetic	Fibrosis and CKD experimental models	DNA methylation reversal, Klotho/protective gene expression	CKD/fibrosis	Genome-wide demethylation, hematological toxicity, oncogenic risk	Preclinical only for renal use (103, 105)
NAD+ boosters	Dual mitoepigenetic	Experimental AKI/aging kidney; translational interest	Sirtuin activity, mitochondrial biogenesis, mitophagy	AKI, aging, CKD	Dose, timing, and durable renal endpoint data unclear	Emerging translational candidate (67)
NRF2 activators	Mitochondrial/redox	Preclinical antioxidant rationale; bardoxolone clinical safety signal	Antioxidant gene induction	DKD/CKD	Cardiovascular adverse events and patient selection risks	Clinically cautionary despite mechanistic appeal (44, 46)
RNA therapeutics	Epigenetic/post-transcriptional	Animal miRNA silencing models	miRNA/pathway modulation, fibrosis reduction	DKD/CKD/fibrosis	Delivery, stability, renal targeting, immune activation	Specific but delivery-limited (76, 95)
Mitochondrial transplantation	Mitochondria-restorative	Mostly non-renal or early experimental models	Bioenergetic restoration	Severe injury hypotheses	Compatibility, engraftment, durability, immune effects	Conceptual/emerging; renal evidence immature (106)

## DNMT inhibitors

DNMT inhibitors have proven to be very promising as antifibrotic and anti-inflammatory drugs used in animal models of kidney diseases. Azacytidine and decitabine, for example, interfere with DNMTs and therefore can help reprogram the gene expression of the protective genes that participate in antioxidant defense and mitochondrial function (107).

In preclinical experiments, the inhibition of DNMT has been shown to reduce the effects of fibrosis induced by TGF-β, decrease inflammation by blocking inflammatory cytokine production, and promote survival of the tubular epithelium (27). Re-establishment of Klotho gene expression along with suppression of fibrotic pathways has also been noted in response to DNMT inhibitors in preclinical models of CKD.

However, in spite of promising results in experimental studies, a number of translational obstacles need to be addressed before any clinical application is considered. DNMT inhibitors lack tissue specificity and cause genome-wide epigenetic changes, which makes them unsuitable for use in clinical settings due to potential risks such as chromosomal instability and hematological toxicities.

## HDAC inhibitors

Histone deacetylase inhibitors have become another promising drug option to reverse kidney diseases through the correction of epigenetic alterations. Various histone deacetylase inhibitors such as vorinostat, trichostatin A, valproic acid, and entinostat alter chromatin accessibility and block inflammatory and fibrotic transcription factor networks (105).

Histone deacetylase inhibitors have been shown to alleviate oxidative stress, antagonize the TGF-β/Smad pathway, and preserve mitochondrial function in experimental animal models of AKI and CKD (103). For instance, trichostatin A prevents fibrosis by blocking the myofibroblastic activation and extracellular matrix deposition while valproic acid decreases the production of inflammatory cytokines and tubular apoptosis (22). In addition, the administration of histone deacetylase inhibitors may also help to improve mitochondrial bioenergetics due to restoration of PGC-1α signaling.

In this respect, targeted sirtuin activation has also emerged as an alternative treatment method that can modulate the epigenome through the modification of histones and, at the same time, maintain mitochondrial homeostasis via deacetylation of NF-κB, p53, and PGC-1α. However, the exact impact of HDAC inhibitors is still unknown as there is evidence showing various physiological roles of HDAC isoforms in renal tissues (104).

## Mitochondria-targeted therapies

Approaches directed at mitochondrial dysfunction are attractive because renal bioenergetics and redox control are disrupted in several kidney diseases. MitoQ has reduced tubular mitochondrial injury and defective mitophagy in experimental diabetic kidney disease, whereas SS-31 (elamipretide) stabilizes cardiolipin and mitochondrial cristae in ischemic models (102). These findings support kidney-specific preclinical activity, but durable clinical renal-endpoint evidence remains limited.

NRF2 activation can enhance endogenous antioxidant defenses, including HO-1, NQO1, and SOD2, but clinical experience with bardoxolone methyl demonstrates that pathway plausibility does not guarantee cardiovascular safety (44, 46). AMPK and SIRT1/PGC-1α activation may improve metabolic adaptation and mitochondrial homeostasis in experimental renal injury; however, the bioavailability and clinical consistency of resveratrol and related SIRT1-directed strategies remain important limitations (31, 90).

Mitophagy modulation is another potential therapeutic approach. Experimental activation of PINK1/Parkin- or BNIP3-associated pathways can improve removal of damaged mitochondria and reduce tubular injury (40, 88). Nevertheless, mitophagy is time- and context-dependent; excessive or persistent autophagic flux during severe injury may worsen energy depletion or interfere with tissue repair (108).

Mitochondrial transplantation remains a proof-of-concept strategy supported mainly by non-renal experimental models, and its relevance to kidney disease is therefore extrapolative (89). NAD+ restoration has more direct renal mechanistic support in experimental AKI and aging, but dose, timing, patient selection, and durable renal outcomes remain unresolved (67).

## Combination therapeutic approaches

Because mitochondrial dysfunction and epigenetic remodeling can reinforce one another, combination approaches are mechanistically plausible. However, superiority over single-pathway therapy has not been established clinically, and combinations may increase off-target effects. Priority should therefore be given to rational, biomarker-guided combinations supported by disease-stage- and cell-specific evidence. Potential combination strategies targeting mitochondrial and epigenetic pathways are illustrated in **Figure 7**.

**Figure 7:**
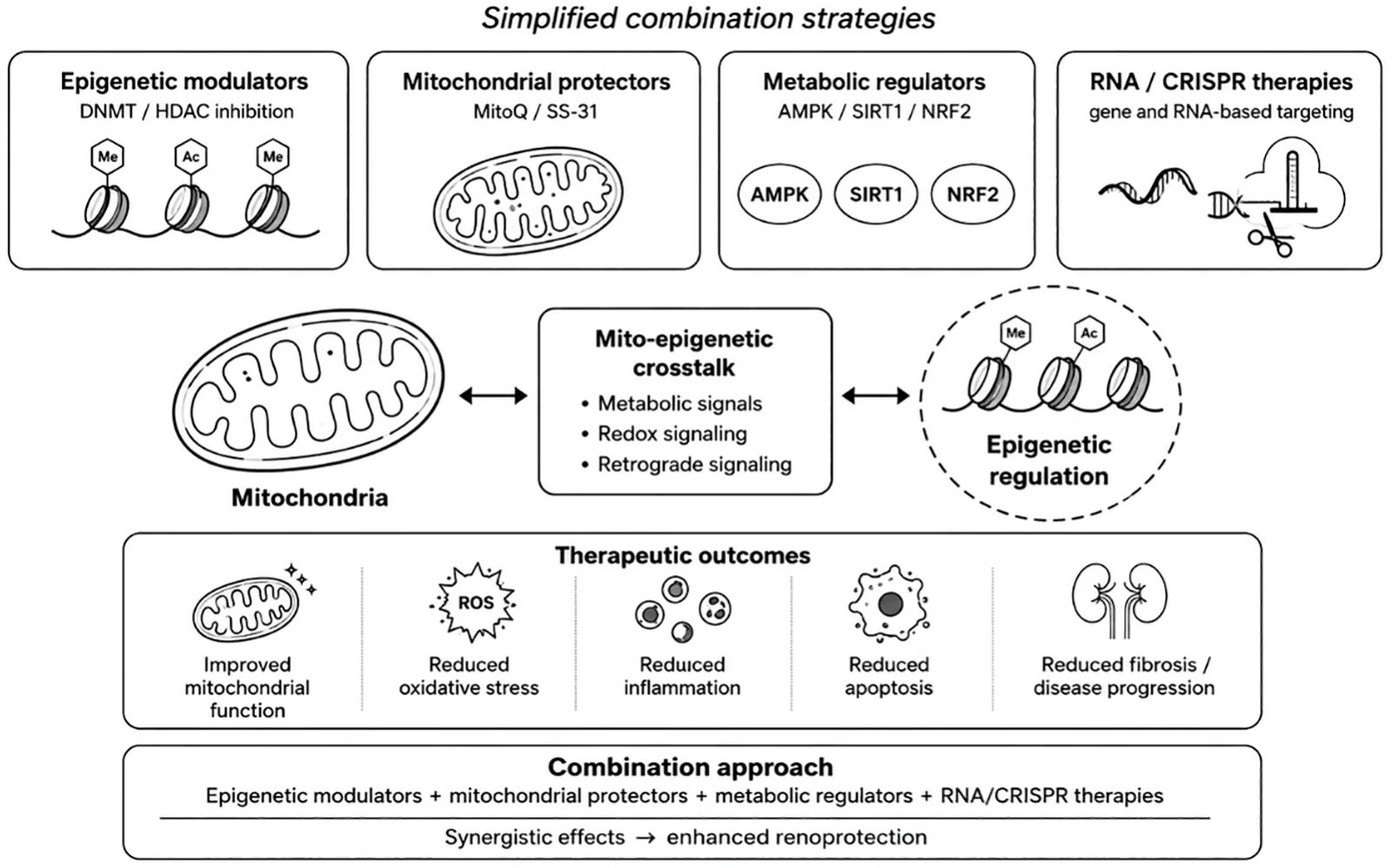
Therapeutic targeting of the mito-epigenetic axis in kidney disease.

Experimental strategies that combine metabolic restoration with selective epigenetic modulation may simultaneously address bioenergetic failure, inflammatory transcription, and fibrotic persistence. Candidate combinations include AMPK/SIRT1 support with isoform-selective epigenetic modulation or NRF2-directed redox control with cell-targeted antifibrotic therapy. These concepts remain preclinical and require formal comparison against single interventions, pharmacokinetic assessment, and renal safety testing (22, 31, 37, 38, 77, 84, 85, 103).

## Challenges and future perspectives

Although mito-epigenetic research in kidney disease has advanced, clinical utility remains limited. A major obstacle is the non-specificity of many epigenetic therapies. DNMT and pan-HDAC inhibition can alter transcription in multiple organs and cell lineages, creating hematological, oncogenic, developmental, or metabolic risks (104). Renal-cell targeting and isoform-selective modulation are therefore central translational requirements.

Kidney-disease heterogeneity is an additional obstacle. AKI, DKD, progressive CKD, aging kidney, and PKD have distinct temporal windows, metabolic states, immune phenotypes, and dominant renal cell populations. Patient-level variation in genotype, comorbidity, mitochondrial reserve, chromatin accessibility, senescence, and medication exposure may further alter therapeutic response. This heterogeneity helps explain why effects observed in a single model may not translate across renal diseases.

Incomplete knowledge of temporal dynamics and cell specificity remains a critical limitation. Bulk-tissue analyses can conceal opposing programs in proximal tubules, podocytes, endothelial cells, fibroblasts, macrophages, lymphocytes, and neutrophils. Single-cell, single-nucleus, spatial, and lineage-tracing approaches can resolve these differences, but technical dissociation bias and the gap between association and causation must be addressed (86).

Precision-nephrology approaches that integrate multi-omics, interpretable computational analysis, and targeted delivery may help prioritize mito-epigenetic interventions (86). Renal-targeted nanoparticles can improve proximal-tubule delivery in experimental systems (109), and mesenchymal-stromal-cell-derived extracellular vesicles have reduced kidney inflammation in experimental models, illustrating a potential delivery platform rather than an established mitoepigenetic treatment (110). CRISPR-based epigenome editing provides a general proof of principle for locus-selective chromatin modulation (111, 112). These technologies remain constrained by delivery efficiency, off-target activity, immunogenicity, durability, safety, and ethical considerations.

Mitochondrial transfer may eventually offer a route to restore cellular bioenergetics, but current evidence is mainly non-renal and should not be presented as established kidney therapy (89). Compatibility, tissue delivery, cellular uptake, organelle integration, durability, and immune consequences require kidney-specific investigation.

Future therapeutic development will require biomarker-based patient stratification. Urinary or circulating mtDNA, KIM-1, NGAL, miR-21, and tissue SIRT1/PGC-1α-related measures provide different information about injury, mitochondrial disruption, fibrosis, or metabolic resilience (67, 76, 95). None is a stand-alone mito-epigenetic biomarker; panels must be validated against disease stage, cell source, treatment response, and durable renal endpoints. The major biomarkers associated with mitochondrial and epigenetic dysfunction and their current limitations are summarized in **Table 6**.

**Table 6:** Biomarkers associated with mitochondrial and epigenetic dysfunction.

Biomarker	Biological meaning	Disease context	Clinical use status	Limitation
mtDNA copy number/urinary mtDNA	Mitochondrial disruption and DAMP burden	AKI, CKD, hypertension-associated renal injury	Translational/clinical association	Cell source and specificity remain uncertain (113)
KIM-1	Proximal tubular injury and maladaptive repair	AKI, CKD, diabetes progression	Clinical/translational	Injury marker rather than direct mitoepigenetic marker (114)
NGAL	Acute tubular injury and inflammatory activation	AKI	Clinical biomarker evidence	Limited specificity for mitoepigenetic mechanism (115)
miR-21	Fibrosis-associated post-transcriptional regulator	DKD, CKD, fibrosis	Experimental/clinical association	Causality and therapeutic delivery remain model-dependent (76, 95)
SIRT1/PGC-1α	Mitochondrial biogenesis and metabolic resilience	AKI, DKD, aging kidney	Experimental/translational	Sampling and cell-specific interpretation are difficult (67)

Accordingly, future research should prioritize renal-cell-targeted therapies, explicit evidence grading, disease-stage stratification, and renal endpoints that distinguish mechanistic biomarker change from durable clinical benefit. Such an approach may help slow progressive nephron loss and fibrosis without overstating the readiness of currently preclinical mito-epigenetic strategies. The principal translational challenges and recommended research priorities are summarized in **Table 7**.

**Table 7:** Challenges and translational priorities.

Challenge	Why it matters	Recommended direction
Disease heterogeneity	AKI, DKD, CKD, aging, and PKD have different timing and dominant cells	Stratify models and patients by disease stage, cell type, and metabolic signature
Evidence grading	Associative omics data are often discussed with causal language	Use intervention-based evidence for causal claims and label hypotheses clearly
Immune complexity	Renal inflammation involves macrophages, T cells, DCs, neutrophils, and resident immune states	Use systems immunology and spatial methods to map immune-renal interactions
Drug specificity	Systemic epigenetic drugs can produce broad off-target effects	Develop renal-cell-targeted delivery and isoform-selective modulators
Clinical endpoints	Improved biomarkers may not predict durable renal benefit	Prioritize eGFR slope, albuminuria, fibrosis endpoints, safety, and patient selection

## Conclusion

Current evidence supports mitochondrial dysfunction and epigenetic remodeling as interdependent components of kidney-disease pathogenesis. Oxidative stress, bioenergetic failure, inflammatory signaling, chromatin remodeling, and profibrotic transcription can form reinforcing loops that contribute to tubular injury, podocyte loss, endothelial dysfunction, maladaptive repair, and renal fibrosis in AKI, CKD, and DKD.

Mitochondrial metabolites and stress signals can influence chromatin-modifying enzymes, while epigenetic programs can alter mitochondrial biogenesis, dynamics, mitophagy, and substrate use. This reciprocal framework extends beyond conventional hemodynamic and glycemic mechanisms, but the strength and direction of causality differ among cell types, disease stages, and experimental models.

Despite substantial translational hurdles, selective modulation of DNMTs, HDACs, mitochondrial ROS, mitophagy, SIRT1/PGC-1α, AMPK, and NRF2 remains scientifically relevant. Precision phenotyping, single-cell and spatial approaches, mitochondrial profiling, and renal-targeted delivery may help identify interventions capable of delaying rather than assuming reversal of progressive renal damage.

Overall, disease- and cell-stratified manipulation of mitochondrial and epigenetic pathways represents a promising research direction for precision nephrology, but clinical adoption will require kidney-specific efficacy, safety, biomarker validation, and durable renal outcome data.
